# Cancer screening in people with intellectual disability: a scoping review of participation, barriers, facilitators, and improvement strategies within the EUCanScreen initiative

**DOI:** 10.1186/s12939-026-02965-1

**Published:** 2026-08-05

**Authors:** Carmen Koko, Zining Yang, Mari Nygård, Patrick Martens, Eliane Kellen, Christoph Kowalski

**Affiliations:** 1 Department of Health Services Research, German Cancer Society, Berlin, Germany; 2 Cancer Registry of Norway, Norwegian Institute of Public Health, Oslo, Norway; 3 Centre for Cancer Detection, Bruges, Belgium; 4 Campus St Rafael, University Hospital Leuven, Leuven, Belgium

**Keywords:** Intellectual disability, Cancer screening, Screening uptake, Barriers, Facilitators, Health equity, Scoping review

## Abstract

**Background:**

People with intellectual disability (ID) experience significant health inequities, yet evidence on their participation in cancer screening and access to preventive care remains fragmented. Conducted within the EUCanScreen Joint Action, this scoping review synthesised global evidence to characterise the current landscape of cancer screening for people with ID and identify approaches to support more inclusive screening practices.

**Methods:**

Following Joanna Briggs Institute methodology and PRISMA-ScR guidelines, we systematically searched MEDLINE, Embase, and Web of Science (2013–2024) for qualitative, quantitative, and mixed-methods studies, supplemented by grey literature. Eligible studies examined participation, barriers, facilitators, or interventions related to six cancers prioritised in European screening policy: breast, cervical, colorectal, prostate, gastric, and lung. Perspectives of people with ID, caregivers, and health care professionals were included. Data were charted and synthesised descriptively, including pathway-based mapping of barriers and facilitators, narrative and visual summaries of screening uptake, and narrative synthesis of intervention studies.

**Results:**

Of 4,387 records screened, 78 met the inclusion criteria. Most studies addressed breast cancer screening (*n* = 53), cervical screening (*n* = 44), and colorectal cancer screening (*n* = 25), which benefit from long-standing organised screening programmes in many countries. In contrast, evidence on prostate cancer screening (*n* = 3) and gastric cancer screening (*n* = 1) was sparse, and no studies examined lung cancer screening, reflecting the far more limited implementation of organised screening for these cancers during the study period. Across contexts, people with ID consistently showed lower participation than the general population. Barriers included fear, inaccessible information, and insufficient provider training, while facilitators involved trusted accompaniment, accessible communication, and tailored examination settings. Intervention studies were scarce but suggested potential for educational, organisational, and tailored support strategies to improve accessibility, preparedness, and participation.

**Conclusion:**

Persistent, multi-level barriers hinder equitable cancer screening for people with ID. Tailored, person-centred screening pathways, improved provider education, and enhanced data systems are needed. Coordinated national and EU-level initiatives, such as EUCanScreen, are vital for structural improvements. Future research should prioritise multicomponent intervention development, pathway-level accessibility, and the inclusion of underrepresented groups to strengthen equity in cancer prevention.

**Supplementary Information:**

The online version contains supplementary material available at 10.1186/s12939-026-02965-1.

## Introduction

Early cancer detection through organised screening programmes is recommended by the World Health Organization (WHO) [1], as it plays a crucial role in reducing the burden of disease and preventing avoidable deaths. Despite decades of organised cancer screening in Europe and worldwide, substantial inequalities in participation persist [2]. These inequalities occur both between countries, reflecting variation in screening policy, programme organisation, implementation, monitoring, and performance [2, 3], and within countries, where participation may differ by socioeconomic position, education, ethnicity, migration background, geographic location, disability, age, and gender-related factors [3–9]. Recognising these challenges, Europe’s Beating Cancer Plan, launched in 2021, emphasises the need for a new EU-supported Cancer Screening Scheme to improve access, quality, and diagnostics [10]. The Council Recommendation (2022/C 473/01) on strengthening prevention through early detection reaffirms organised population-based screening for breast, cervical, and colorectal cancer, and invites Member States to explore the feasibility and stepwise implementation of organised screening for lung and prostate cancer, as well as for gastric cancer in regions with high incidence and mortality [11]. In line with this prioritisation, the present review focuses on these six cancer entities: breast, cervical, colorectal, lung, prostate, and gastric cancer.

Importantly, organised population-based screening programmes are not uniformly implemented across these cancers. While breast cancer screening, cervical screening, and colorectal cancer screening have long been established in many countries, organised screening for lung, prostate, and gastric cancer remains limited, is restricted to selected countries or high-risk populations, or is still under evaluation [12]. Accordingly, empirical evidence on cancer screening is more readily available for cancers and countries with mature, organised programmes and routine monitoring systems, whereas evidence remains limited where screening is newer, less organised, restricted to pilots or selected populations, or not systematically monitored [3].

Within this policy context, the EUCanScreen Joint Action (2024–2028) brings together 29 European countries to support the implementation of high-quality, equitable cancer screening [13]. Conducted within the framework of the Joint Action, this scoping review aims to raise awareness of cancer screening in people with intellectual disability (ID) and provide evidence to guide future programme development.

Intellectual disability is defined by the American Association on Intellectual and Developmental Disabilities (AAIDD) as “significant limitations both in intellectual functioning and adaptive behaviour” originating before the age of 22 [14]. This definition aligns closely with that of the Diagnostic and Statistical Manual of Mental Disorders, Fifth Edition (DSM-5), which also emphasises limitations in intellectual and adaptive functioning with onset during childhood [15]. A meta-analysis of 52 population-based studies estimated the global prevalence of ID at 10.37 per 1,000 population [16]. However, this estimate is likely to be conservative, given that people with mild ID frequently remain undiagnosed and are consequently underrepresented in administrative and registry-based data [17].

People with ID require specific consideration in health care provision. The United Nations Convention on the Rights of Persons with Disabilities (Article 25) obliges Member States to ensure equal access to health care, including preventive services, for people with disabilities [18]. Despite improvements leading to increased life expectancy over recent decades, the lifespan of people with ID remains substantially shorter than that of the general population [19], accompanied by poorer overall health [20, 21] and lower utilisation of health care services [22]. These inequalities are embedded in broader challenges in health care provision for people with ID, including unmet health needs, barriers to timely diagnosis and treatment, communication difficulties, dependence on family or paid carers for advocacy, and insufficient reasonable adjustments in health care settings [23]. Evidence from mortality research further indicates an over-representation of potentially avoidable deaths among adults with ID, suggesting gaps in prevention, early detection, and appropriate care [24]. Furthermore, evidence shows that cancers are often diagnosed at more advanced stages in people with ID, contributing to higher mortality rates compared to those without ID [25, 26]. Recent publications in The Lancet Oncology and The Lancet Public Health have further drawn attention to the persistent neglect of people with ID in cancer screening and outcomes, underscoring the growing recognition of this issue in leading medical discourse [27, 28].

In this context, equitable cancer screening should not be understood as maximising participation at all costs, but as ensuring accessible, informed, and person-centred decision-making. Participation in screening is voluntary and may involve potential harms, including false-positive results, unnecessary follow-up or treatment, and screening-related stress, pain, or embarrassment [29]. However, evidence suggests that people with ID are often insufficiently supported to make informed and autonomous decisions about screening [30].

Previous reviews have explored factors influencing cancer screening participation among people with ID. One systematic review focused specifically on barriers and facilitators to screening participation, identifying obstacles such as fear and distress, negative interactions with health care professionals, lack of knowledge, and mobility issues, while also highlighting facilitators such as supervised living environments and prior engagement with health services [31]. Evidence on cancer outcomes and disparities across the entire continuum, not limited to screening, has also been synthesised and reports consistent inequalities across the cancer care continuum for people with intellectual and developmental disabilities (IDD) [32]. Additional reviews examined specific cancer types, broader disability populations, or individual countries [33–36]. One systematic review found that women with ID are significantly less likely to undergo cervical screening compared to women without [33], while another UK-based review emphasised the importance of adequate support and accessible information to enable informed participation in breast cancer and cervical screening [34]. A recent synthesis further focused on educational interventions, drawing on eight studies that aimed to improve cancer awareness and screening among people with ID [37].

Overall, research addressing cancer screening in this population remains limited across countries, and evidence on effective strategies to improve screening uptake is particularly scarce. While existing reviews have advanced understanding, they have generally focused on individual cancer types, specific national contexts, broader disability populations, educational interventions, or cancer-related disparities beyond screening. A broader synthesis is therefore needed that brings together evidence across cancer entities, stakeholder perspectives, and stages of the screening pathway. The present scoping review addresses this gap by synthesising global evidence across breast, cervical, colorectal, prostate, gastric, and lung cancer screening and by integrating findings on screening participation, barriers, facilitators, and interventions from the perspectives of three key stakeholder groups: people with ID, their caregivers, and health care professionals. This comprehensive approach provides a holistic overview of the worldwide cancer screening landscape for people with ID, helps identify evidence gaps across cancer entities and screening pathway stages, and informs the design of more inclusive and equitable screening programmes.

### Research questions

The aim of this review is to answer the following questions:

1. What is the current state of research on cancer screening (screening rates, barriers and facilitators) among people with ID and what are the main findings?
2. What approaches have been elaborated and/or tested in empirical research to improve cancer screening for people with ID?

## Methods

### Design and reporting framework

This scoping review was conducted following the Joanna Briggs Institute (JBI) methodology for scoping reviews [38] and is reported in accordance with the PRISMA-ScR (Preferred Reporting Items for Systematic Reviews and Meta-Analyses extension for Scoping Reviews) checklist [39]. An a priori protocol was registered on the Open Science Framework platform [40].

### Eligibility criteria

We applied the PCC (Population, Concept, Context) framework [38] to define our inclusion criteria:

- **Population**: People with ID, explicitly including studies using the broader term of IDD [41]. We also considered studies involving paid or unpaid caregivers and health care professionals caring for this population. In collaboration with two experts in health in people with ID, we identified relevant codes from the International Classification of Diseases, 10th Revision (ICD-10) to capture the target population (see Supplementary Material S1 for the complete list). Heterogeneity in population terminology across studies is described in the Results section.
- **Concept**: Empirical studies (qualitative, quantitative, or mixed-methods) that either:

- Describe the current cancer-screening landscape for people with ID, covering participation rates, barriers, and facilitators (Concept 1); or
- Develop, test, or evaluate strategies, interventions, policies, and programmes aimed at improving any aspect of the screening pathway (e.g. accessibility, invitation processes, modality adaptations, provider training, communication, and patient experience) (Concept 2).

- **Context**: All health care settings worldwide where cancer screening is offered or studied.

No language restrictions were applied. We excluded studies that:

- Focused broadly on developmental, cognitive, or other types of disabilities without providing differentiated results for people with ID;
- Addressed diagnostic or therapeutic aspects of cancer without screening outcomes;
- Were non-empirical (e.g., editorials, opinion pieces), reviews, case reports, or conference records without sufficient empirical information for data extraction.

Conference records were eligible only if they provided sufficient empirical information for data extraction. Where potentially eligible conference records were available only as abstracts, authors were contacted to clarify whether a full publication or sufficiently detailed material was available. If a corresponding peer-reviewed full publication was identified, only the full publication was included to avoid duplicate reporting.

### Search strategy

We conducted comprehensive searches in MEDLINE (via Ovid), Embase, and Web of Science for publications dated May 2013 through early December 2024, reflecting the time point immediately after the DSM-5 release and its updated definition of ID [15]. Each database search combined controlled vocabulary (MeSH in MEDLINE; Emtree in Embase) with synonyms and free-text terms for “intellectual disability” and “cancer screening,” linked with Boolean operators. The complete search strategies for MEDLINE, Embase, and Web of Science are provided in Supplementary Material S2–S4. To ensure breadth, we also hand-searched grey literature (governmental reports, key organisational websites) and screened reference lists of relevant reviews.

### Study selection

**Fig. 1:**
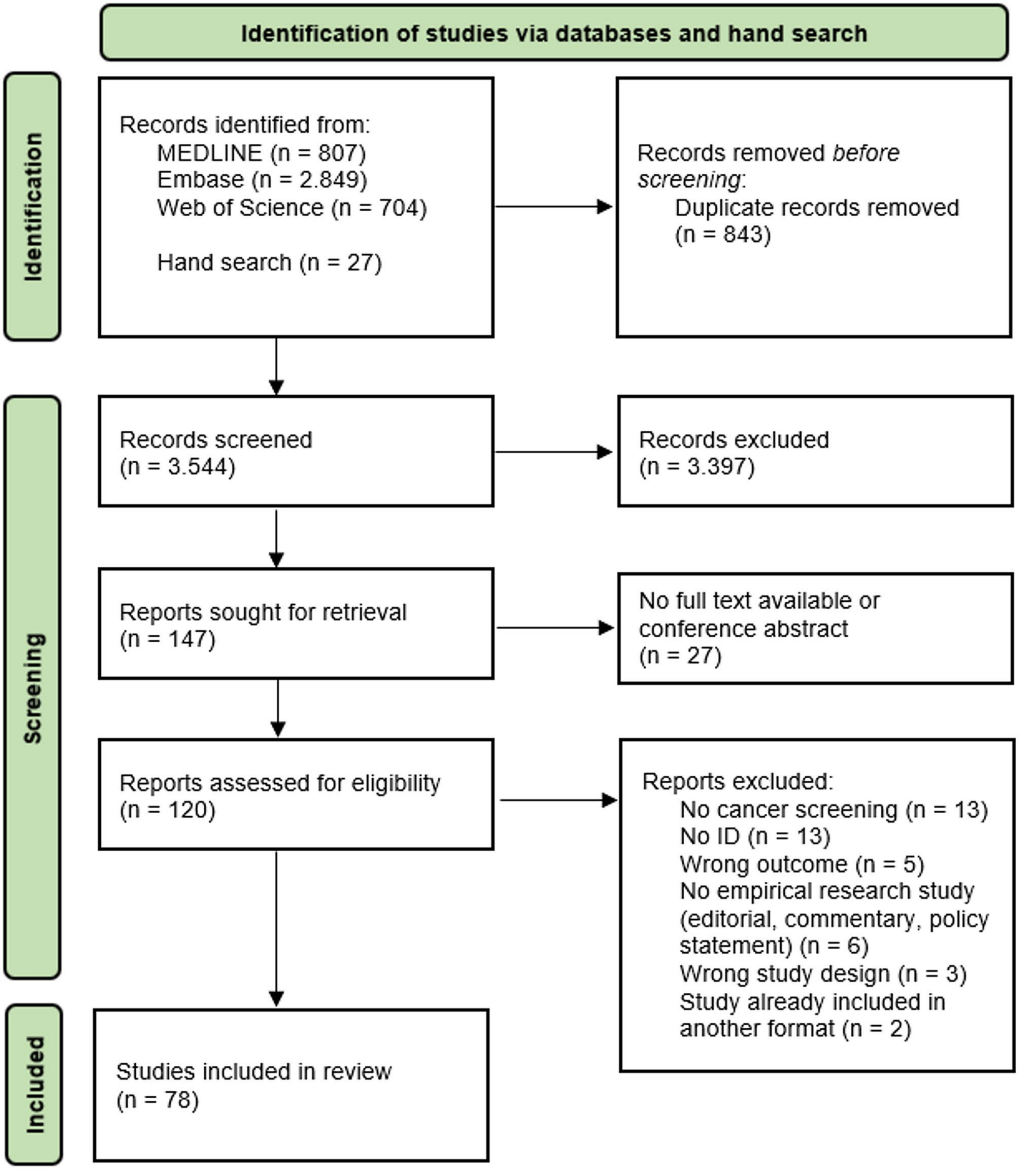
PRISMA flow diagram for inclusion of studies [42]

All records were imported into Rayyan, where potential duplicate records were identified using automated similarity matching and subsequently reviewed and resolved manually by the first author (CaKo). Two reviewers (CaKo, CK) independently screened titles and abstracts against the eligibility criteria. Discrepancies at the title and abstract screening stage were resolved through discussion between the two reviewers. Full texts of potentially relevant studies were then retrieved and independently assessed in duplicate by two reviewers (CaKo, ZY); any discrepancies were resolved through discussion or, when needed, consultation with a third reviewer (CK). Reasons for full-text exclusions were recorded and are presented in the PRISMA flow diagram (Fig. 1) in the Results section.

### Data extraction

Using a JBI-inspired extraction template [38], two reviewers independently extracted the following from each included study: author(s), year, country, study design, cancer entities, objectives, population, concept, context, type of source, and main findings. Disagreements were settled by consensus, and study authors were contacted to clarify or supplement missing information when necessary. The complete data extraction table, including all charting fields and extracted study-level information, is provided in Supplementary Material S5.

### Data analysis and synthesis

Data analysis unfolded in two main phases and was led by the first author (CaKo), with regular discussion and validation of analytic decisions within the author team. First, we profiled the included studies, summarising their designs, geographic distribution, targeted cancer types, populations, and conceptual focus both narratively and with summary graphics. Descriptive summaries were generated from the extracted study-level data. Second, we structured our synthesis around the two core concepts:

1. Screening participation evidence

- Screening rates: We charted quantitative uptake data and assessed whether studies were suitable for graphical comparison based on the presence of an internal comparison group, a defined screening interval, and a clearly reported sample size for people with ID. Studies that did not meet these criteria were retained in the narrative synthesis to ensure comprehensive coverage of the evidence.
- Barriers and facilitators: Findings on barriers and facilitators were extracted from the included studies and mapped to the initial steps of the WHO screening pathway, as outlined in the WHO Regional Office for Europe guide on cancer screening [29]. The pathway stages used for organisation were: (1) Identification of the target population, (2) Invitation and information, (3) Testing, (4) Referral of screen positives and reporting of screen-negative results, and cross-pathway factors. The extraction and pathway-based organisation of barriers and facilitators were conducted by the first author and discussed within the author team to resolve uncertainties and ensure consistency.

2) Strategies and attempts for improvement
  While facilitators refer to contextual or structural factors observed to support screening participation, strategies and attempts for improvement describe concrete interventions that were tested or proposed to enhance screening uptake. We synthesised these strategies, including awareness initiatives, provider training, and alternative screening methods, into a concise narrative of improvement efforts.

### Deviations from protocol

Although our protocol initially referred to “screening experiences,” we refined this to focus explicitly on “barriers and facilitators” to provide clearer analytic categories.

### Ethical considerations

As this review used only published and publicly available data, no ethical approval was required.

## Results

### Search results

Database and hand searches yielded 4,387 records. After screening and eligibility assessment, 78 studies were included in the review. The study selection process is shown in Fig. 1.

### Study characteristics

The study characteristics are summarised in Table 1. The complete data extraction table, including the objectives and key findings of each study, is provided in Supplementary Material S5.

**Table 1:** Study characteristics of included studies

Author and year	Country	Cancer entity	Study design	Population	Concept*
Agaronnik et al. 2020 [43]	USA	Cervical	Qualitative (interviews/ focus groups)	Physicians	1)
Arana et al. 2019 [44]	USA	Breast	Quantitative (observational)	Women with ID, self-identifying as Black, Hispanic, White	1)
Arana-Chicas et al. 2020 [45]	USA	Breast	Qualitative (interviews)	Women with ID, caregivers	1)
Armin et al. 2022 [46]	USA	Breast, cervical	Qualitative (interviews)	Native American women with IDD, caregivers, providers, community members/ leaders	1/2)
Bae et al. 2022 [47]	UK	Cervical	Quantitative (observational)	Women who are non-attenders in cervical screening (subgroup analysis for women with LD)	1/2)
Bates, Triantafyllopoulou 2019 [30]	UK	Breast	Quantitative (observational)	Women with ID	1)
Bourgarel et al. 2016 [48]	France	Colorectal	Quantitative (observational)	Adults with ID	1)
Bowel Cancer UK 2019 [49]	UK	Colorectal	Quantitative (interventional)	Health and social care professionals	2)
Bowler, Nash 2015 [50]	UK	Colorectal	Quantitative (interventional)	Adults with LD	2)
Breau et al. 2020 [51]	Canada	Breast, colorectal	Quantitative (observational)	Primary care providers and trainees	1)
Breau et al. 2023 [52]	Canada	Breast, cervical, colorectal	Qualitative (interviews)	Family physicians and family medicine trainees	1)
Brown HK et al. 2016 [53]	Canada	Cervical	Quantitative (observational)	Women who had a pregnancy with IDD and without IDD	1)
Brown M et al. 2016 [54]	USA	Breast, cervical, colorectal	Quantitative (observational)	Adults with ID	1)
Chen et al. 2023 [55]	Taiwan	Cervical	Quantitative (observational)	Adults with disabilities (subgroup analysis for adults with IDD) and without disabilities	1)
Chen et al. 2024 [56]	USA	Cervical	Quantitative (observational)	Women with autism spectrum disorder (subgroup analysis for women with ID)	1)
Cithambaram et al. 2023 [57]	Ireland	Breast, cervical, colorectal	Qualitative (interviews/ focus groups)	Adults with ID, health care professionals, family carers	1)
Cobigo et al. 2013 [58]	Canada	Breast, cervical	Quantitative (observational)	Women with IDD and without IDD	1)
Collins et al. 2014 [59]	UK	Breast	Qualitative (interviews)	Women with ID, health and social care professionals, managers and staff from voluntary and statutory services, carers	1)
Conder et al. 2019 [60]	New Zealand	Breast, cervical	Qualitative (interviews)	Health clinicians and disability support service staff	1)
Deroche et al. 2017 [61]	USA	Colorectal	Quantitative (observational)	Adults with ID and comparison group	1)
Durbin et al. 2019 [62]	Canada	Breast, cervical, colorectal	Quantitative (observational)	Adults with IDD	2)
Folch et al. 2019 [63]	Spain	Breast, cervical	Quantitative (observational)	Adults with intellectual developmental disorders	1)
Glover et al. 2014 [64]	UK	Breast, cervical, colorectal	Quantitative (observational)	Adults with LD	1)
Gray et al. 2021 [65]	UK	Colorectal	Quantitative (interventional)	Adults with LD, carers	2)
Greenwood et al. 2014a[66]	USA	Breast	Qualitative (interviews)	Family members of women with ID	1)
Greenwood et al. 2014b [67]	USA	Breast	Quantitative (interventional)	Women with ID	2)
Haider et al. 2013 [68]	Australia	Breast, cervical, colorectal	Quantitative (observational)	Adults with ID and the general Victorian population	1)
Havercamp, Scott 2015 [69]	USA	Breast, cervical, prostate	Quantitative (observational)	Adults with IDD, other disabilities, and without disabilities	1)
Horsbøl et al. 2023 [70]	Denmark	Breast	Quantitative (observational)	Women with ID and without ID	1)
Hu et al. 2023 [71]	Sweden	Cervical	Quantitative (observational)	Women with mental illness, psychiatric or neurodevelopmental disorders (subgroup analysis for women with ID)	1)
Hughes-McCormack et al. 2021 [72]	UK	Cervical	Quantitative (observational)	Women with ID and women from the general population	1)
Humphrey et al. 2021 [73]	USA	Breast	Quantitative (observational)	Women with Rett syndrome	1)
Inchai et al. 2022 [74]	Taiwan	Breast	Quantitative (observational)	Women with disabilities (subgroup analysis for women with ID) and without disabilities	1)
Jensen et al. 2021 [75]	USA	Breast, cervical, colorectal	Quantitative (observational)	Adults with Down syndrome	1)
Kellen et al. 2020 [76]	Belgium	Breast, cervical, colorectal	Quantitative (observational)	Adults with ID	1)
Khan et al. 2024 [77]	USA	Breast	Quantitative (observational)	Adults with IDD and without IDD	1)
Kim et al. 2020 [78]	South Korea	Gastric	Quantitative (observational)	Adults with disabilities (subgroup analysis for adults with ID) and without disabilities	1)
Lai et al. 2014 [79]	Taiwan	Breast	Quantitative (observational)	Women with ID	1)
Lee et al. 2024 [80]	USA	Cervical	Qualitative (interviews)	Primary care providers	1)
Limbaugh et al. 2021 [81]	USA	Cervical	Quantitative (observational)	Young women with developmental delay (Down syndrome, Rett syndrome, and chromosomal translocations)	2)
Lloyd, Coulson 2014 [82]	UK	Cervical	Qualitative (interviews)	Learning disability nurses	1)
Maltais et al. 2020 [83]	Canada	Breast, cervical, prostate	Quantitative (observational)	Adults with ID	1)
Marquis et al. 2023 [84]	Canada	Cervical	Quantitative (observational)	Women with IDD and without IDD	1)
McCarron et al. 2023 [85]	Ireland	Breast, cervical, colorectal	Mixed-methods (cohort study with questionnaires and interviews)	Adults with ID	1)
Mirfin-Veitch et al. 2016 [86]	New Zealand	Breast, cervical	Qualitative (interviews)	Women with LD, disability and health professionals	1)
Miyashita et al. 2024 [87]	Japan	Breast	Quantitative (observational)	Women with severe motor and intellectual disabilities	2)
Olsen et al. 2023 [88]	Norway	Breast, cervical	Quantitative (observational)	Women with ID	1)
Otandault et al. 2024 [89]	France	Breast, cervical, colorectal	Quantitative (interventional)	Adults with ID	2)
Ouellette-Kuntz et al. 2015a [90]	Canada	Breast, cervical, colorectal	Quantitative (observational)	Adults with IDD and without IDD	1)
Ouellette-Kuntz et al. 2015b [91]	Canada	Colorectal	Quantitative (observational)	Adults with IDD and without IDD	1)
Parish et al. 2013 [92]	USA	Cervical	Quantitative (observational)	Women with ID	1)
Plourde et al. 2018 [93]	Canada	Breast, cervical	Quantitative (observational)	Women with ID	1)
Reidy et al. 2018 [94]	Ireland	Breast	Quantitative (observational)	Women with mild to moderate ID	1)
Ricour et al. 2022 [95]	Belgium	Breast, cervical	Quantitative (observational)	Women with ID	1)
Schieron et al. 2023 [96]	Germany	Colorectal	Mixed-methods (focus groups/ interviews and questionnaires)	Adults with ID, medical staff, researcher	2)
Shin et al. 2018 [97]	South Korea	Cervical	Quantitative (observational)	Women with disabilities (subgroup analysis for women with ID) and without disabilities	1)
Shin et al. 2020a [98]	South Korea	Colorectal	Quantitative (observational)	Adults with disabilities (subgroup analysis for adults with ID) and without disabilities	1)
Shin et al. 2020b [99]	South Korea	Breast	Quantitative (observational)	Women with disabilities (subgroup analysis for women with ID) and without disabilities	1)
Smith et al. 2020 [100]	USA	Breast, cervical	Quantitative (observational)	Women with Down syndrome	1)
Son et al. 2013 [101]	USA	Breast, cervical	Mixed-methods (interviews, chart review)	Women with ID	1)
Stokes et al. 2023 [102]	USA	Breast, colorectal	Quantitative (observational)	Adults with IDD	1)
Swaine et al. 2013 [103]	USA	Breast, cervical	Qualitative (interviews)	Female family caregivers	1)
Swaine et al. 2014 [104]	USA	Breast, cervical	Quantitative (interventional)	Women with ID	2)
Sykes et al. 2022 [105]	UK	Breast, cervical	Qualitative (Q methodology)	Women with LD, family carers, and paid care workers	1)
Sykes et al. 2024 [106]	UK	Breast, cervical	Qualitative (triangulated study)	Women with LD, family carers, and paid care workers	1/2)
Trétarre et al. 2017 [107]	France	Breast	Quantitative (observational)	Women with ID	1)
Turner et al. 2013 [108]	UK	Breast, cervical, colorectal	Qualitative (interviews)	Adults with LD	1 /2)
Walsh et al. 2022 [109]	Ireland	Breast	Qualitative (interviews, focus groups)	Women with mild/moderate ID, caregivers, and health care professionals	2)
Wang et al. 2015 [110]	USA	Breast	Quantitative (observational)	Women with ID	1/2)
Weise et al. 2024 [111]	Australia	Breast	Mixed-methods (modified Delphi study using Likert-scale ratings + thematic analysis)	Experts with backgrounds in health care provision, accessible screening services, and disability advocacy	2)
Wellkamp et al. 2022 [112]	Germany	Breast, cervical, colorectal, prostate	Quantitative (observational)	Adults with ID	1)
Willis et al. 2015 [113]	UK	Breast	Qualitative (ethnographic approach: observation, interviews)	Paid carers, family carers	1)
Willis et al. 2016 [114]	UK	Breast	Qualitative (interviews, focus group observations)	Women with ID	1)
Wood et al. 2024 [115]	USA	Breast, colorectal	Quantitative (observational)	Adults with Down syndrome	1)
Wyatt, Talbot 2013 [116]	UK	All cancer types	Quantitative (observational)	Paid carers	1)
Xu et al. 2017 [117]	USA	Breast, cervical	Quantitative (observational)	Women with ID	1)
Yen et al. 2014 [118]	Taiwan	Cervical	Quantitative (observational)	Women with ID	1)
Zeilinger et al. 2024 [119]	Austria	Colorectal	Mixed-methods (interviews/ focus groups, questionnaires)	Adults with ID, caregivers	1)

*Empirical studies (qualitative, quantitative, or mixed-methods) that either:

1) Describe the current cancer-screening landscape for people with ID—covering participation rates, barriers, and facilitators; or

2) Develop, test, or evaluate strategies, interventions, policies, and programmes aimed at improving any aspect of the screening pathway

Abbreviations: ID = intellectual disabilities; IDD = intellectual and developmental disabilities; LD = learning disabilities

#### Study design and cancer entities

A total of 78 studies were included in the analysis [30, 43–119]. Quantitative designs were most common (*n* = 55), predominantly observational, with a smaller number of interventional studies [30, 44, 47–51, 53–56, 58, 61–65, 67–79, 81, 83, 84, 87–95, 97–100, 102, 104, 107, 110, 112, 115–118]. Qualitative approaches were used in 18 studies, primarily interview-based designs [43, 45, 46, 52, 57, 59, 60, 66, 80, 82, 86, 103, 105, 106, 108, 109, 113, 114], while five studies applied mixed-methods approaches [85, 96, 101, 111, 119]. Most records were peer-reviewed journal articles (*n* = 69) [30, 43–48, 50–56, 58–84, 87, 88, 90–94, 97–107, 109–118], while nine were grey literature sources [49, 57, 85, 86, 89, 95, 96, 108, 119]. These included research, evaluation, and institutional reports, as well as one conference poster that provided sufficient empirical information on an awareness intervention and extractable results. Reporting detail varied across grey literature sources, particularly regarding methodological information and sample-size reporting. Many studies addressed more than one cancer type. Breast cancer screening was examined most frequently (*n* = 53) [30, 44–46, 51, 52, 54, 57–60, 62–64, 66–70, 73–77, 79, 83, 85–90, 93–95, 99–115, 117], followed by cervical (*n* = 44) [43, 46, 47, 52–58, 60, 62–64, 68, 69, 71, 72, 75, 76, 80–86, 88–90, 92, 93, 95, 97, 100, 101, 103–106, 108, 112, 117, 118] and colorectal cancer screening (*n* = 25) [48–52, 54, 57, 61, 62, 64, 65, 68, 75, 76, 85, 89–91, 96, 98, 102, 108, 112, 115, 119]. Prostate cancer screening was addressed in three studies [69, 83, 112], gastric cancer screening in one study [78], and no study specifically focused on lung cancer screening. One study addressed cancer screening comprehensively [116].

#### Population

Across the 78 included studies, all three stakeholder groups (people with ID, their caregivers, and health care professionals) were included as study populations, with some studies focusing exclusively on one population and others including multiple groups. People with ID were the most frequently studied population, appearing in 66 studies [30, 44–48, 50, 53–59, 61–65, 67–79, 81, 83–102, 104–110, 112, 114, 115, 117–119]. Among studies including people with ID, sample sizes ranged from *n* = 3 to *n* = 86 in qualitative studies and from *n* = 4 to *n* = 49,114 in quantitative studies. Caregivers, both paid and unpaid, were included in 17 studies [45, 46, 49, 57, 59, 60, 65, 66, 86, 103, 105, 106, 109, 113, 116, 119], with sample sizes ranging from *n* = 2 to *n* = 32 in qualitative studies and from *n* = 28 to *n* = 324 in quantitative studies. Health care professionals were included in 14 studies [43, 46, 49, 51, 52, 57, 59, 60, 80, 82, 86, 96, 109, 111], with sample sizes ranging from *n* = 2 to *n* = 42 in qualitative studies and from *n* = 106 to *n* = 304 in quantitative studies. Sample sizes refer to the respective stakeholder subgroup within each study where available, rather than necessarily to the total study population, and may include overlapping participants across publications. Detailed study-level sample size information is provided in Supplementary Material S5.

The terminology used to describe the study population varied across studies, including “intellectual disabilities” (*n* = 49) [30, 43–45, 48, 51, 52, 54, 56, 57, 59–61, 66–68, 70–72, 74, 76, 78, 79, 82, 83, 85, 88, 89, 92–99, 101, 103, 104, 107, 109–114, 117–119], “intellectual and developmental disabilities” (*n* = 12) [46, 53, 55, 58, 62, 69, 77, 80, 84, 90, 91, 102], and “learning disabilities” (*n* = 10) [47, 49, 50, 64, 65, 86, 105, 106, 108, 116]. A smaller number of studies focused on specific populations, including people with Down syndrome (*n* = 3) [75, 100, 115], people with Rett syndrome (*n* = 1) [73], and people with “severe motor and intellectual disabilities” (*n* = 1) [87]. Less frequently used terms included “developmental delay” (*n* = 1) [81] and “intellectual developmental disorders” (*n* = 1) [63]. During the screening process, it was verified that the included studies reported results for people with ID as defined by the AAIDD and met the predefined inclusion and exclusion criteria. As described in the Methods section, although IDD represents a broader conceptual category that may include conditions such as autism spectrum disorder, ID constitutes a core component of this definition [41, 120]; studies using this terminology were therefore included. For reasons of readability, the term ID is used throughout the Results section, acknowledging heterogeneity in population definitions across studies.

#### Geographical distribution

**Fig. 2:**
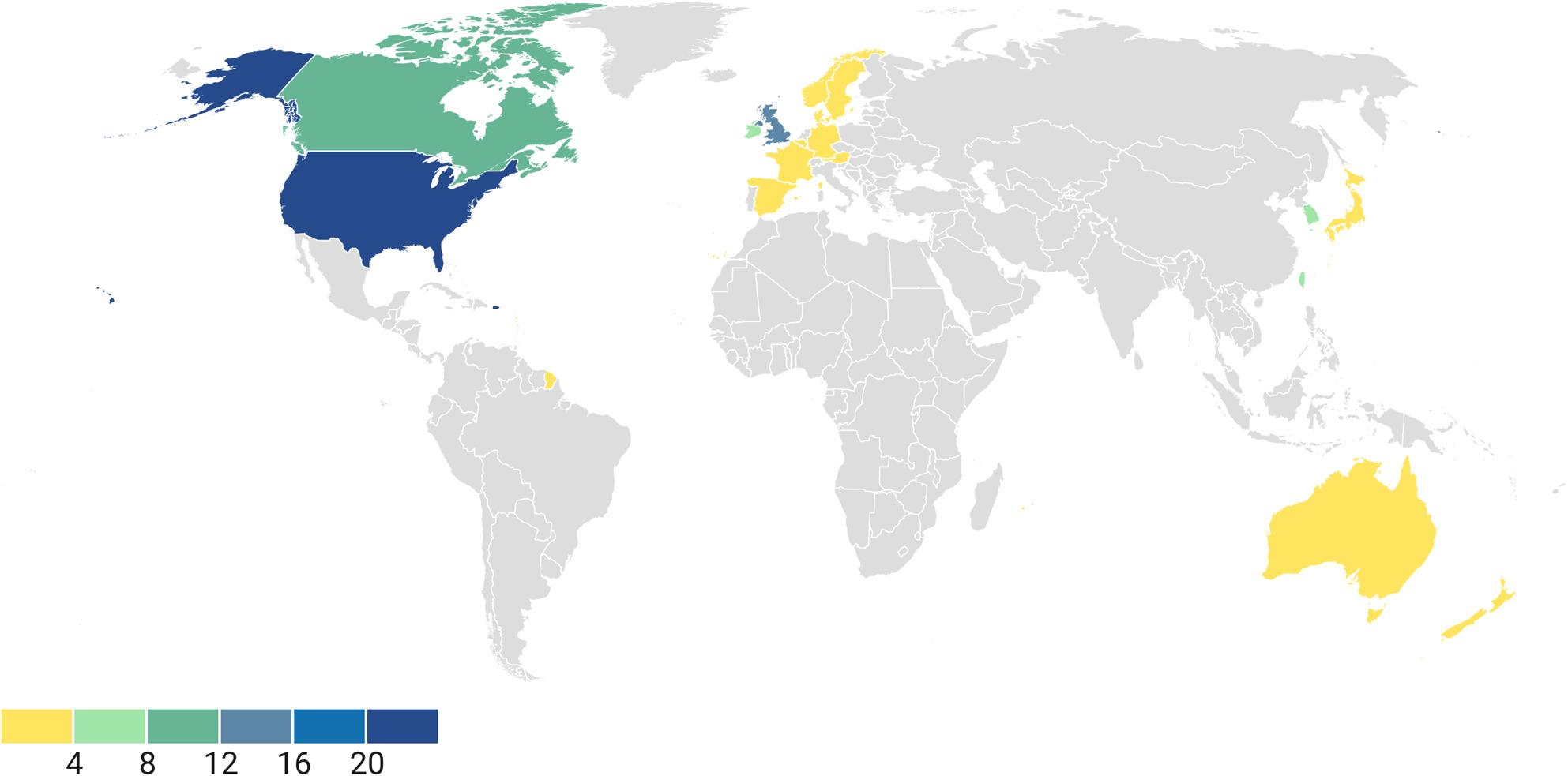
Geographical distribution of included studies on cancer screening in people with ID

All included studies were conducted within a single-country context; no international or cross-country studies were identified. In total, the studies originated from 16 different countries. Most studies were conducted in the United States (USA) [43–46, 54, 56, 61, 66, 67, 69, 73, 75, 77, 80, 81, 92, 100–104, 110, 115, 117] (*n* = 24), followed by the United Kingdom (UK) [30, 47, 49, 50, 59, 64, 65, 72, 82, 105, 106, 108, 113, 114, 116] (*n* = 15) and Canada [51–53, 58, 62, 83, 84, 90, 91, 93] (*n* = 10). Several studies also came from Ireland [57, 85, 94, 109], South Korea [78, 97–99], Taiwan [55, 74, 79, 118] (*n* = 4), and France [48, 89, 107] (*n* = 3). Single or few studies were identified from countries such as Australia [68, 111], Belgium [76, 95], Germany [96, 112] and New Zealand [60, 86] (*n* = 2), Austria [119], Denmark [70], Japan [87], Norway [88], Spain [63] and Sweden [71] (*n* = 1). An overview of the geographical distribution of the included studies is presented in Fig. 2.

#### Conceptual focus

Most studies examined the current state of cancer screening in people with ID (Concept 1; *n* = 61), investigating participation, screening rates, barriers, and facilitators [30, 43–45, 48, 51–61, 63, 64, 66, 68–80, 82–86, 88, 90–95, 97–103, 105, 107, 112–119]. Studies evaluating interventions, pilot projects, or programme evaluations aimed at improving screening practices were less common (Concept 2; *n* = 12) [49, 50, 62, 65, 67, 81, 87, 89, 96, 104, 109, 111]. A small number of studies combined elements of both research questions (*n* = 5) [46, 47, 106, 108, 110].

### Concept 1: Screening participation evidence

#### Barriers and facilitators organised according to the initial stages of the WHO screening pathway [29]

##### Stage 1: Identification of the target population

In most screening programmes, eligibility is defined by demographic criteria such as age and sex, meaning that people with ID are, in principle, included in the target population. However, one report from New Zealand highlights that, for the National Cervical Screening Programme, eligibility is based not only on age but also on sexual activity, making it less straightforward to determine which women should be enrolled [86]. Screening registries generally do not capture disability status, which prevents identifying people who may require specific communication or support measures. One British report noted that women with learning disabilities were not identified in the NHS Cervical Screening Programme’s Call and Recall System, meaning that invitation and result letters could not routinely be provided in accessible formats [108]. Facilitators included structural mechanisms that improved the systematic identification of people with ID within screening-eligible populations. For breast screening, one British report described that Breast Screening Services invited women based on lists compiled from GP records and could, since mid-2012, flag women with ID in the National Breast Screening System (NBSS) when notified by Public Health or local disability services. This could support the recording of reasonable adjustments and the inclusion of ID as a variable in reports extracted from the NBSS [108].

##### Stage 2: Invitation and information

Invitation and information processes varied across countries and screening programmes, including direct invitations from screening programmes or call-and-recall systems, registry-based communication, and varying degrees of involvement of caregivers, residential services, and primary care providers. These differences are relevant because people with ID may require accessible formats, additional explanation, or support to understand invitations and make informed decisions about participation. Significant barriers arose from inadequate knowledge, inexperience, and limited awareness concerning the benefits of cancer screening or the needs of people with ID in medical contexts among caregivers, family members, medical professionals, and people with ID themselves [43, 45, 46, 52, 59, 62, 80, 82, 94, 103, 105, 113, 116, 119]. Negative attitudes held by medical staff and caregivers [51, 57, 82], misconceptions or unawareness about sexual activity in women with ID regarding cervical screening [43, 60, 80, 85, 105], and unclear or inaccessible information further hindered participation [57, 59, 66, 86, 105, 108]. Difficulties related to the severity of ID, decision-making support needs, and uncertainty around guardianship [30, 43, 52, 57, 60, 66, 80, 113] further complicated this stage, as did the exclusion of people with ID from decision-making processes [30, 113].

Facilitating factors were collaborative decision-making and effective interdisciplinary cooperation [60, 82, 106], targeted preparatory measures (e.g., desensitisation sessions and pre-visits) [45, 66, 82, 86, 103, 105], clear, accessible information (e.g., Easy Read formats, visuals), and effective communication practices [43, 45, 60, 86, 105, 106, 108, 119]. Additionally, in some cases mammography was perceived by family members as a symbol of adult womanhood, sexuality, and societal inclusion (‘sisterhood’), as one qualitative study revealed [66].

##### Stage 3: Testing

Testing procedures varied across cancer screening programmes and settings. Clinic-based procedures included, for example, mammography and clinician-collected cervical screening, while colorectal cancer screening was often described as home-based stool testing, with colonoscopy commonly required as follow-up after a positive test result. These different testing modalities shaped the types of barriers reported. Clinic-based procedures and follow-up examinations were particularly associated with fear, anxiety, pain, discomfort, and embarrassment [45, 46, 52, 66, 85, 94, 103, 105, 113]. For home-based colorectal cancer screening using stool tests, available evidence pointed to the need for accessible instructions and support to understand the procedure, collect the sample where needed, and complete the test process [57, 108]. Limited family or social support [45, 66, 113] and structural barriers such as inappropriate facilities, inflexible scheduling, time constraints, lack of personalised accommodations, and inadequate transportation posed additional significant obstacles [43, 45, 46, 52, 57, 59, 60, 82, 108, 113, 119]. Communication difficulties with the medical staff [30, 43, 46, 59, 66, 80, 113] and past negative health care experiences also discouraged participation [114].

Facilitators included empathetic, trained personnel [45, 59, 82, 86, 108, 119], utilisation of supportive methods (e.g., sedation or adapted screening methods) [43, 45], flexible scheduling and extended examination times [60, 86, 105], transportation access [102], active, well-prepared and supportive caregivers [45, 46, 60, 66, 86, 103, 113], and a positive attitude towards screening in people with ID [45], as well as the involvement of female practitioners particularly in cervical and breast cancer screening [105].

##### Stage 4: Referral of screen positives and reporting of screen-negative results

The fear of subsequent examinations following a positive screening result and a possible decline in quality of life was mentioned in one study as a potential barrier [66]. Processes for reporting screening results and arranging follow-up varied across screening programmes and settings, including direct communication with the screened person, communication via GPs, or involvement of caregivers and support persons. Few studies addressed the communication of screening outcomes and follow-up processes in detail. However, available evidence suggests that results are often shared primarily with caregivers or GPs rather than directly with the people who were screened, thereby limiting person-centred feedback [57]. Facilitators included direct communication of results to the screened people [60] and the opportunity to review the results together [108].

##### Cross-pathway factors

Poor coordination of care and unclear responsibilities were identified as substantial systemic barriers across all stages of the screening process [45, 57, 59, 105, 106]. Lower screening rates and awareness were noted among people living independently or with families compared to structured residential settings [44, 92, 94, 112, 117]. Financial worries and a preference for traditional medicine further hindered screening participation [46]. Living in socially vulnerable neighbourhoods also negatively influenced screening uptake [77].

**Fig. 3:**
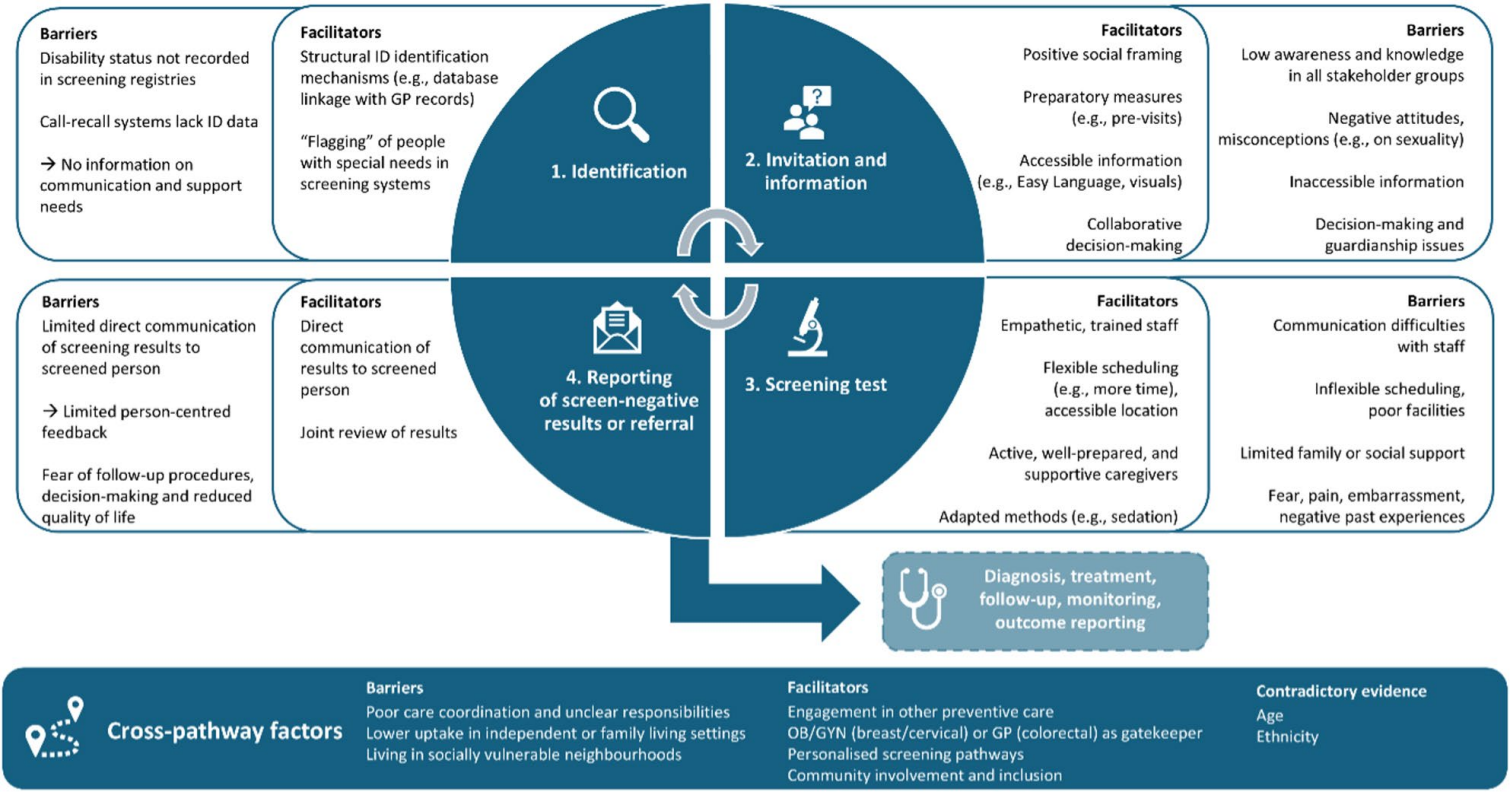
Barriers and facilitators across the initial stages of the cancer screening pathway for people with ID

Factors positively influencing participation included approaches that can be understood as personalised screening pathways, combining accessible information, service coordination, individualised reasonable adjustments, and trusted support across the screening process [60, 66, 86, 108, 119]. Other facilitators included community involvement and community inclusion [102], prior personal or familial cancer experience [79, 94, 118], engagement with other preventive health care services [79], rural residency [92, 98, 118], being married [79, 118], higher educational levels [79], and higher income [118]. Enrolment in a primary care physician–based model for colorectal cancer screening [91], and care provided by specialists such as obstetricians and gynaecologists rather than GPs for cervical and breast cancer screening, were found to facilitate screening uptake [43, 92, 93]. Evidence regarding ethnicity was mixed. Aboriginal status in a Taiwanese study [118] and Black or Hispanic ethnicity in one US mammography study [44] were associated with higher screening uptake or frequency, whereas a large US Medicare-based study [77] found lower odds of breast cancer screening among non-Hispanic Black and Hispanic individuals. Evidence regarding age was also mixed. While some studies reported higher uptake with older age, particularly for colorectal cancer screening and mammography [88, 90, 91], others found lower participation with increasing age, including cervical screening in Taiwan [118] and colorectal cancer screening in South Korea [98]. Figure 3 illustrates how identified barriers and facilitators align with the initial steps of the screening pathway.

#### Screening rates

##### Study heterogeneity

**Fig. 4:**
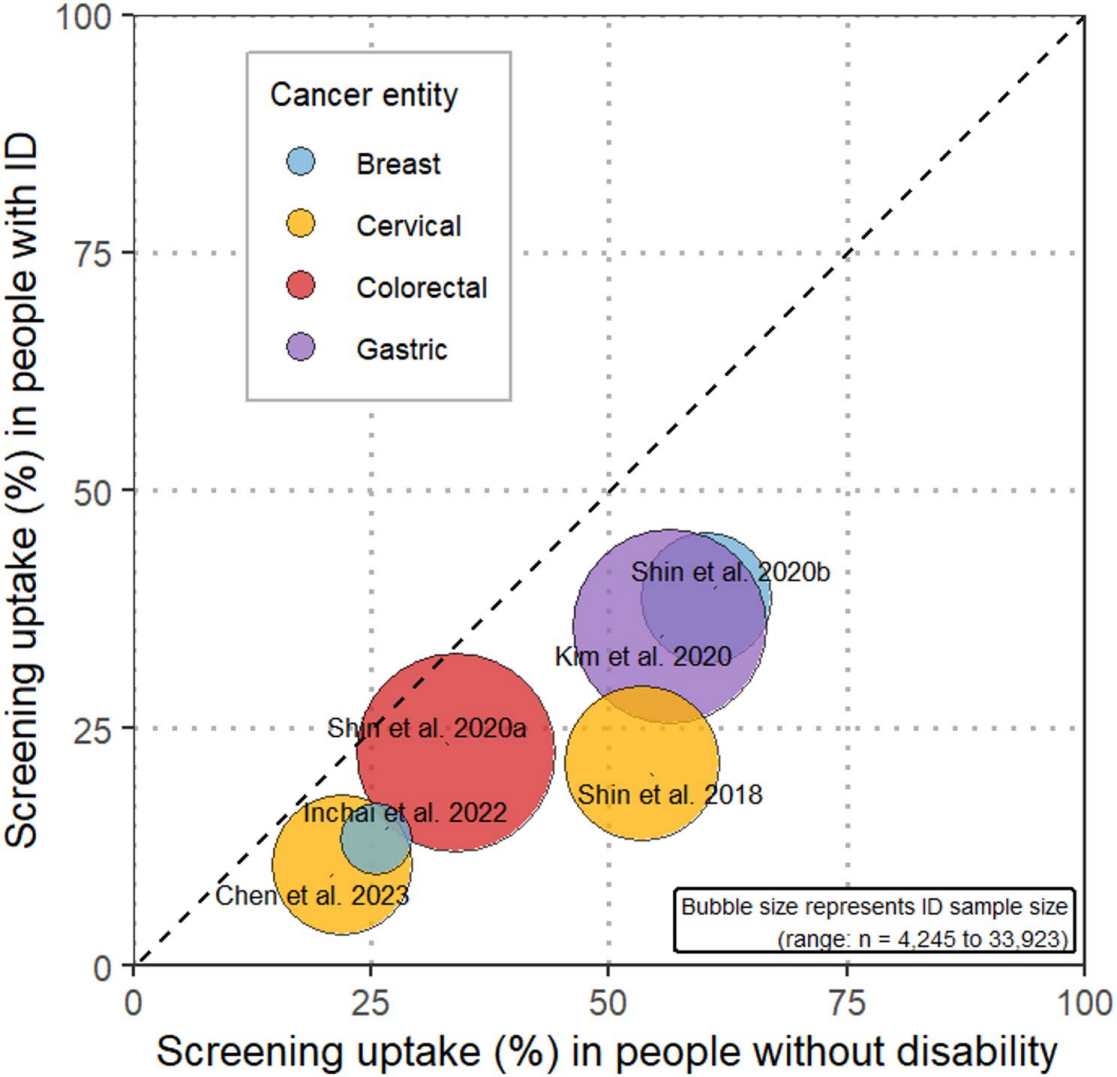
Comparison of cancer screening uptake between people with ID and without disability (per national recommendations)

**Fig. 5:**
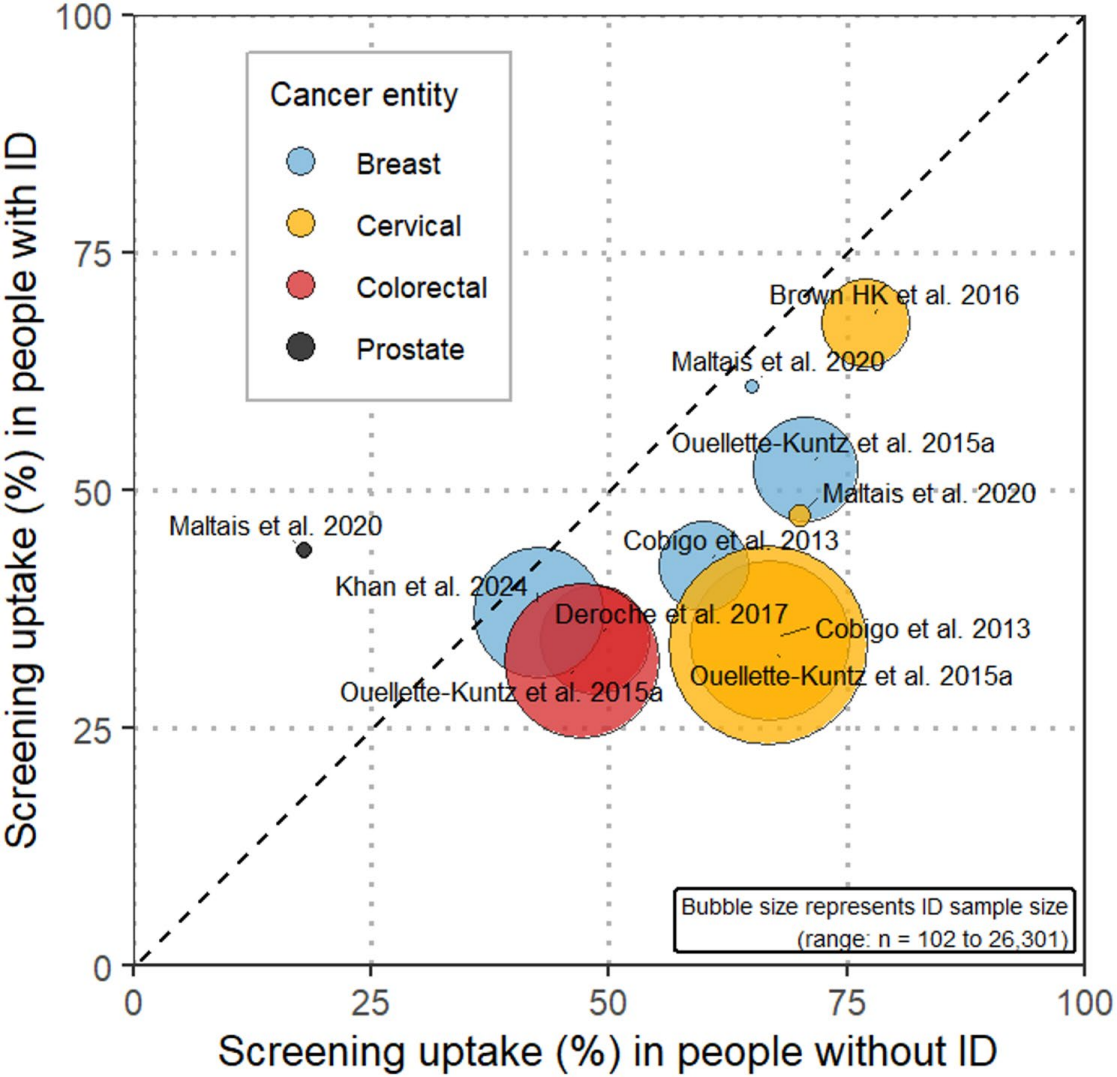
Comparison of cancer screening uptake between people with and without ID (per national recommendations)

The studies reporting screening rates varied widely in design, reporting, study populations, and outcome definitions, which precluded direct comparison of most results. To enable meaningful synthesis, we restricted the graphical comparison to studies that (1) included a coherent internal comparison group, either contrasting people with ID and people without ID (Fig. 4) or analysing ID as one subgroup among several disabilities and therefore using people without disability as the reference group (Fig. 5); (2) reported screening uptake within a defined national screening interval; and (3) provided a clearly defined sample size (n) for people with ID for a specific cancer entity.

Twelve studies met these conditions, with six comparing people with ID with people without ID [53, 58, 61, 77, 83, 90] and six comparing people with ID with people without disability [55, 74, 78, 97–99], permitting the graphical summaries shown in Figs. 4 and 5. All remaining studies reporting screening rates are included in the narrative synthesis below to ensure a comprehensive overview of the available evidence.

Those studies were either designed with different internal comparison groups, relied on national programme benchmarks rather than an internal comparison group, or did not report the sample size of the ID subgroup in sufficient detail. Five studies examined related outcomes such as adherence over multiple screening cycles [70, 117], the effect of continuity of primary care [93], or the influence of capacity level [30], and race/ethnicity [44] on screening uptake.

##### Findings on screening rates

Although methodologies varied, most studies consistently found that people with ID had lower screening rates than comparison groups or national benchmarks. This trend was observed across multiple cancer screening types. Lower participation in cervical screening was reported in 24 studies [53, 55, 56, 58, 63, 64, 68, 69, 71, 72, 75, 76, 83–85, 88, 90, 92, 95, 97, 100, 112, 117, 118]. Similarly, reduced uptake for breast cancer screening was documented in 19 studies [58, 63, 64, 68–70, 74–77, 79, 83, 88, 90, 95, 99, 100, 112, 117]. Lower participation was also reported for colorectal cancer screening [48, 61, 64, 68, 75, 76, 90, 91, 98, 112], gastric cancer screening [78], and prostate cancer screening [69, 112].

Conversely, some studies reported higher screening uptake among populations with ID compared to the general population, in some cases based on very small study populations, subgroup analyses, or clearly defined settings such as supported living facilities. This applies to four studies on breast cancer screening [54, 85, 107, 112], one on prostate cancer screening in people with moderate-to-profound ID [83], one on cervical screening [54], and two on colorectal cancer screening [54, 85].

##### Severity of ID and screening rates

Eight studies stratified their analyses by severity of ID, with six reporting that screening rates typically decreased with increasing severity [44, 70, 76, 79, 83, 118]. However, two studies found no significant association with severity [88, 92], and one study reported increasing rates with severity for colorectal cancer screening [76]. Furthermore, several studies observed that screening rates decreased with increasing disability severity across various disability types [55, 74, 78, 97–99]. Additionally, one study highlighted poorer overall health status with increasing severity of ID [63]. Overall, the available evidence suggests an association between greater severity of ID and lower screening uptake, but the findings were not fully consistent across studies.

### Concept 2: Strategies and attempts for improvement

Some studies (*n* = 17) developed, tested, or evaluated strategies, interventions, policies, or programmes aimed at improving cancer screening uptake and preparedness among people with ID, across different cancer types and care settings, with generally promising findings [46, 47, 49, 50, 62, 65, 67, 81, 87, 89, 96, 104, 106, 108–111].

#### Less invasive methods

The use of ultrasonography alone for breast cancer screening in women with severe motor and intellectual disabilities residing in long-term care facilities in Japan has been examined over a seven-year period, demonstrating effectiveness with low recall rates and suggesting suitability as a minimally invasive alternative for highly dependent populations [87]. Similarly, blind Pap tests performed without a speculum have been assessed as a less invasive cervical screening option for young women with developmental delays. Despite a lower detection rate of certain cell types, the tests demonstrated 100% specimen adequacy, indicating clinical utility when traditional exams are not tolerated [81]. This evidence refers specifically to cytology-based Pap testing, although human papillomavirus (HPV) testing is now the primary screening method in many settings.

#### Raising awareness and knowledge…

##### …In people with ID

Efforts to increase awareness and knowledge among people with ID have shown promising results. An interventional study in France involving adults with mild to moderate ID demonstrated significant improvements in cancer screening knowledge three months after the intervention (*p* = 0.0012), as well as greater increases in intentions to participate in screening in the intervention group (+ 1.1 points) compared with controls (+ 0.7 points) [89]. One-year follow-up results were not available at the time this manuscript was prepared. A DVD-based intervention in the USA demonstrated moderate improvements in mammography preparedness among women with mild to moderate ID, with mean preparedness scores increasing from 3.8 to 4.2 (out of 5) following the intervention, alongside improvements in knowledge of mammography purpose and procedures [67]. A randomised controlled trial in the USA evaluated *Women Be Healthy* and its revised version, *Women Be Healthy 2*, structured health education curricula for women with ID addressing breast and cervical cancer screening [104]. The original programme comprised eight weekly 90-minute sessions and used multimodal, hands-on activities, including breast models, videos showing screening procedures, practice in communicating with health professionals, exposure to medical settings, and anxiety-reduction techniques. The revised version comprised 11 twice-weekly 60-minute sessions, expanded the content on cervical cancer, Pap tests, and pelvic anatomy, and added further repetition, discussion, resource materials, and skills-building activities. Overall, the revised intervention was associated with modest improvements in screening-related knowledge, including appropriate action when detecting a breast lump, understanding of the Pap test, Pap test picture identification, and strategies to reduce anxiety during examinations. The mean number of correct responses increased from 3.7 to 5.4 out of nine items following the intervention [104]. In the UK, a targeted 12-week community intervention to boost colorectal cancer screening among adults with ID identified eligible participants, addressed barriers such as inaccessible information and embarrassment, trained staff, and provided support throughout the screening process. As a result, screening uptake increased from 47% to 61% [50]. One German evaluation report described a participatory model project addressing, among other topics, colorectal cancer prevention and early detection for people with ID. The project developed Easy Language information materials, explanatory videos, and a physician training concept on accessible communication. The evaluation focused mainly on participation processes and product development rather than screening uptake. It included surveys, focus groups, interviews, and pilot testing with people with ID, physicians, and other professionals; for example, 157 questionnaires from people with ID and an online survey with 66 physicians informed the development and evaluation of project materials [96].

##### … in health and social care professionals

A UK project report evaluated a colorectal cancer screening training programme for health and social care professionals supporting adults with ID [49]. From March 2018 to January 2019, 22 full-day training sessions were delivered across 11 Scottish health board areas to 304 attendees. The sessions covered colorectal cancer symptoms, risk factors, and screening, and also addressed accessible communication and examples of tailored support to promote informed choice. The evaluation reported a 77% increase in colorectal screening knowledge from pre- to post-training, and all attendees reported feeling confident explaining screening after the training [49]. A Canadian study evaluated a Health Check protocol for adults with ID in two Ontario family health teams, which also aimed to support primary care staff in providing proactive preventive care [62]. The protocol included identifying patients with ID through electronic medical records, proactively inviting them for a Health Check, and conducting a modified health examination supported by point-of-care tools and staff education on ID in primary care. Cancer screening outcomes included Pap testing, mammography, and faecal occult blood testing. Mammography and faecal occult blood testing rates were higher in the Health Check group than in the non-Health Check group, although not significantly so, while Pap testing remained low in both groups. Staff who performed Health Checks reported higher comfort, knowledge, and skills in caring for adults with ID, although gaps in preparedness and resources persisted [62].

#### Further strategies

A modified online Delphi study reached consensus on 52 strategies to improve access to breast screening for people with ID, including strategies related to decision-making and consent, accessible information, peer mentorship, service navigation, trauma-informed care, and equipping key stakeholders [111]. Conversely, short message service reminders alone showed limited effectiveness in increasing cervical screening uptake among women with ID, highlighting the need for more tailored and supportive approaches [47]. A qualitative formative study described the adaptation of an existing breast and cervical cancer screening education programme for Native American women with ID and their caregivers. The adapted programme, *My Health My Choice*, incorporated traditional ways of knowing about health, family and community contexts, dyadic delivery with a chosen caregiver, local health educators known to the community, and links to local spiritual leaders or healers in the event of a diagnosis. The study therefore provided formative evidence for culturally sensitive adaptation rather than outcome data from an implemented intervention [46]. Finally, a local British strategy and toolkit described concrete pathways for improving access to cervical, breast, and colorectal cancer screening for people with ID [108]. Across these cancer screening pathways, key elements included identification of people with ID within screening systems, Easy Read invitations and accessible information, training and support for professionals, support staff, and family carers, reasonable adjustments, guidance on consent and best-interests decision-making, and arrangements for information sharing, result communication, and follow-up [108].

## Discussion

### Main findings and interpretation

This scoping review synthesised evidence from 78 studies on cancer screening participation, barriers, facilitators, and intervention strategies among people with ID. Four main findings emerged. First, people with ID generally showed lower cancer screening participation than people without ID or the general population, although uptake varied considerably by cancer type, country, setting, living arrangement, study design, and individual factors such as age and severity of ID. Second, the evidence base was uneven across both cancer types and geographic regions. Third, barriers and facilitators were distributed across the screening pathway, suggesting that screening inequities are not produced at a single point of access but emerge through the interaction of individual, interpersonal, organisational, and programme-level factors. Fourth, intervention evidence remained limited and fragmented, with most strategies focusing on education, awareness raising, accessible materials, or local support initiatives rather than multicomponent interventions addressing accessibility across the full pathway.

Taken together, these findings suggest that screening inequalities among people with ID should not be interpreted primarily as individual non-attendance or lack of knowledge. Rather, they appear to reflect a mismatch between standardised screening pathways and the communication, decision-making, sensory, relational, and organisational support needs of this population. This interpretation is consistent with broader evidence on health care for people with ID and with general cancer screening literature showing that uptake is shaped by knowledge and health literacy, perceived risk, fear and embarrassment, provider recommendation, practical accessibility, and support in navigating services [121–126].

### Evidence distribution and gaps

Breast cancer screening and cervical screening were the most frequently addressed (*n* = 53 and *n* = 44, respectively), highlighting their significant public health relevance and the fact that both have long-established programmes in many countries [127, 128]. A moderate number of studies focused on colorectal cancer screening (*n* = 25), while prostate and gastric cancer were less commonly examined (*n* = 3 and *n* = 1, respectively). No studies were identified that directly investigated lung cancer screening among people with ID, reflecting the relatively recent or less widespread implementation of organised screening for lung, prostate and gastric cancer. This uneven focus limits conclusions for less-studied cancers, although some barriers may plausibly apply across cancer types.

While most studies directly focused on people with ID (*n* = 66), some also included health care professionals (*n* = 14) and caregivers (*n* = 17), highlighting the influence of multiple stakeholders on screening perception and uptake [34].

The geographic distribution of studies reveals a clear concentration in high-income, English-speaking countries, particularly the USA, the UK, and Canada, whereas evidence from Eastern Europe and from low- and middle-income countries remains scarce. This uneven representation mirrors disparities in health care infrastructure, registry capabilities, and research investment [2, 129]. Moreover, the lack of standardised and comprehensive registries capable of reliably identifying people with ID complicates international efforts to assess screening coverage, reflecting broader challenges in quantifying cancer burden in this population. These gaps underscore the urgent need for expanded data collection and monitoring [130], especially in underrepresented regions.

### Screening participation and heterogeneity

Lower participation in cancer screening programmes among people with ID compared with the general population or national benchmarks was a major finding across the included evidence. This pattern spans multiple cancer types, including breast, cervical, colorectal, gastric, and prostate cancer. However, some studies reported higher screening rates, especially in subpopulations living in supported residential settings [54, 107]. Given the methodological heterogeneity of these studies, including differences in sample size, data sources, comparison groups, and screening intervals, these findings should be interpreted as context-dependent rather than evidence of consistently equitable access. They nevertheless suggest that living arrangements, structured support, and regular contact with health or social care services may positively influence uptake.

This interpretation of participation gaps is consistent with broader evidence on health care for people with ID, which describes barriers related to communication, professional knowledge and attitudes, care coordination, under-recognition of health needs, and the need for appropriate support across health care settings [121–123]. In this context, lower screening uptake may indicate that organised screening programmes include people with ID in principle, but do not consistently provide the support required to make participation accessible in practice.

The reviewed studies confirm that people with ID represent a highly heterogeneous population with diverse health profiles and needs. For instance, Rethoré et al. discussed differentiated screening approaches for adults with Down syndrome, such as clinical breast monitoring instead of routine mammography, based on potentially distinct cancer risk profiles [131]. However, such adaptations are not currently reflected in European screening guidelines. This heterogeneity, coupled with varying terminology, including intellectual disabilities, intellectual and developmental disabilities, intellectual developmental disorders, and learning disabilities, and the inclusion of different diagnostic codes and concepts limits comparability and generalisability. Considerable variation in how ID was identified across studies, ranging from medical records and self-report to registry entries and social programme inclusion, has also been reported in a previous review [32]. Few studies (*n* = 8) stratified outcomes by severity of ID. Overall, the available data suggest that greater severity of ID is generally associated with lower screening uptake [44, 70, 76, 79, 83, 118], although this pattern was not consistent across all studies. Differences in how severity was defined, the cancer screening modality examined, study setting, and data sources may contribute to variation across findings. Further research is required that explicitly accounts for the heterogeneity of ID, including severity and support needs, as such differentiation is essential to inform inclusive, person-centred and tailored preventive care [132].

### Screening barriers in relation to existing evidence

Barriers clustered around identification, communication, professional preparedness, decision-making support, and the testing encounter. These included the lack of identification of ID within health registries, inaccessible or non-tailored information, limited health care professional training, challenges related to decision-making support needs and uncertainty around guardianship, and psychological factors such as fear, previous negative health care experiences, and communication difficulties.

Many of these barriers are also well established in the general cancer screening literature, including fear, embarrassment, limited knowledge, low perceived need, transport difficulties, lack of provider recommendation, and difficulties navigating appointments or services [124–126]. For people with ID, however, these barriers may be intensified because screening programmes often rely on written communication, individual appointment management, abstract risk information, and tolerance of unfamiliar clinical procedures. This suggests that commonly recommended screening strategies need to be adapted to the communication and support needs of people with ID.

### Strategies and implications for practice

Evidence from the general screening literature suggests that interventions addressing only one dimension of screening behaviour may have limited impact when organisational, practical, or trust-related barriers persist [133, 134]. Multicomponent approaches, including provider recommendation, patient navigation, tailored reminders, mailed or home-based testing options, and reduction of logistical barriers, have shown promise in improving screening uptake in underserved populations [133, 134]. The intervention evidence identified in this review points in a similar direction: accessible information and education are important, but are likely to be most effective when combined with proactive support, trained professionals, flexible appointments, and reasonable adjustments.

Efforts to address these barriers are underway. In England, the NHS has introduced a national Reasonable Adjustment Flag within electronic health records to identify people who may require additional support, including those with ID, and to prompt providers to implement appropriate adjustments across health care settings [135]. At the European level, the CUPID project (since 2022) aims to improve understanding of cancer prevention among people with ID by establishing a research agenda and knowledge base across the EU and beyond [136]. Since 2024, the EUCanScreen Joint Action exemplifies coordinated efforts to close data gaps and implement inclusive screening approaches [137]. Within this project, the Turin Memorandum was formulated at a May 2025 expert meeting in Turin and signed by experts from participating countries. It calls for political and structural commitment to equitable cancer screening access for people with ID [138].

Several innovative screening adaptations are currently being explored. In cervical screening, HPV-based testing is now recommended by the WHO and the European Commission Initiative on Cervical Cancer as the preferred primary screening method [139, 140], and several countries have incorporated HPV self-sampling into organised programmes [141]. For people with ID, HPV self-sampling may be acceptable and empowering when accompanied by tailored information and practical assistance [80], and an ongoing feasibility study within EUCanScreen is investigating this approach. Other adaptations, such as blind Pap tests or ultrasound as an alternative to mammography, should be interpreted cautiously because they are not currently part of European population-level screening recommendations [11, 81, 87].

Facilitators that enhance screening uptake include trusted accompaniment, flexible appointment scheduling, accessible communication formats, and training for people with ID, caregivers, and professionals. Resources like the Easyhealth website provide valuable easy-to-read materials in the context of health, including cancer-related content, supporting people with ID, caregivers and health care professionals [142]. Petitpierre et al. recently developed easy-to-read-and-understand brochures on cancer and cancer screening for French-speaking regions, with an evaluation planned [143]. Similarly, in Ireland, pictorial materials, easy-to-read leaflets co-designed with people with ID, and videos have been developed and implemented to facilitate informed participation [144–146].

These findings point to several practical next steps for screening programmes: identifying and documenting support needs; strengthening accessible invitation and decision-support processes; implementing reasonable adjustments during testing, including longer appointments, preparation, trusted accompaniment, and trained staff; and establishing clearer arrangements for result communication and follow-up.

### Strengths and limitations

Strengths of this review include the use of established scoping review methodology, transparent reporting according to PRISMA-ScR, and registration of an a priori protocol. The review provides a broad synthesis across six cancer entities and multiple stakeholder perspectives, integrating evidence on screening participation, barriers, facilitators, and improvement strategies. The structured presentation of screening rates, the mapping of barriers and facilitators across the screening pathway, and the inclusion of grey literature strengthen both the methodological transparency and practical relevance of the synthesis.

This review has several limitations. Restricting the search to studies published between May 2013 and early December 2024 aligns with evolving diagnostic criteria such as the DSM-5, but may have excluded relevant earlier or very recent research. Furthermore, the substantial heterogeneity in defining and identifying ID complicates synthesis and may obscure subgroup-specific trends. Likely underdiagnosis and under-registration, particularly among people with mild ID, together with international variation in health care systems and screening programme structures, further challenge comparability and generalisability.

Grey literature was included to capture practice-based initiatives, programme evaluations, and reports that may not yet be reflected in peer-reviewed publications. However, these sources varied in their aims and reporting detail, particularly regarding methods, sample characteristics, and outcomes. Findings from grey literature should therefore be interpreted mainly as mapping emerging approaches and evidence gaps rather than as robust evidence of effectiveness.

Finally, the search strategy prioritised sensitivity by focusing on a manageable set of key terms and searching three major databases. Consequently, relevant studies may have been missed, particularly those using alternative terminology or published in less accessible sources. Although no language restrictions were applied, the English-language search terms may have led to an underrepresentation of studies published in other languages. To mitigate retrieval bias, reference lists of related reviews, grey literature databases, and national health reports were also screened; however, some relevant evidence may still be absent.

## Conclusion

This review confirms persistent disparities in cancer screening participation among people with ID across multiple cancer types. These disparities reflect not only individual-level barriers, but also limitations in how mainstream health care and organised screening programmes identify, communicate with, support, and accommodate people with ID. Barriers were found across the screening pathway, underscoring the need for pathway-level approaches rather than isolated adjustments.

The heterogeneity among people with ID highlights the need for person-centred screening approaches that account for different support needs, living arrangements, communication preferences, and severity of ID. Emerging strategies, including reasonable adjustments, accessible information, educational interventions, professional training, and adapted screening methods, show potential to improve screening accessibility and participation. However, evidence on comprehensive, multicomponent interventions remains limited.

Future research should prioritise standardised data collection, inclusion of underrepresented populations, and rigorous evaluation of interventions that address accessibility across the full screening pathway. Outcomes should extend beyond uptake to include informed choice, screening experience, acceptability, distress, and completion of follow-up. European initiatives such as the EUCanScreen Joint Action and the CUPID project provide important opportunities to strengthen coordinated policy, research, and practice efforts.

Reducing cancer screening inequalities for people with ID requires screening programmes that move beyond nominal inclusion towards practical accessibility, supported informed decision-making, and equitable preventive care.

## Supplementary Information

Below is the link to the electronic supplementary material.

**Supplementary Material 1:** Supplementary Tables S1–S4

**Supplementary Material 2:** Supplementary Table S5

## Data Availability

All data generated or analysed during this study are included in this published article and its supplementary files.

## Declarations

### Use of artificial intelligence tools

Generative artificial intelligence tools were used solely to support language editing and improve clarity of expression during manuscript preparation. All content was reviewed and approved by the authors. During study selection, the software Rayyan was used to flag potential duplicate records based on automated similarity matching. All duplicates were subsequently reviewed and resolved manually by the reviewers, and no screening or data extraction decisions were automated.
