# Supplementary material for: Cancer screening in people with intellectual disability: a scoping review of participation, barriers, facilitators, and improvement strategies within the EUCanScreen initiative
Source: Int J Equity Health. 2026 Aug 5;25:186. doi: 10.1186/s12939-026-02965-1 (PMC13445881; doi:10.1186/s12939-026-02965-1)
Supplement: Supplementary file 1 [file 12939_2026_2965_MOESM1_ESM.docx]

**Supplementary Material S1-S4**

**Table S1: Population**

According to ICD-10 and in exchange with two experts in this field from Germany and France, we have identified the following relevant diagnosis codes for the presence of ID:

| **Diagnosis** | **ICD-10 Code** |
| --- | --- |
| Intellectual disabilities | F70–F79 |
| Rett syndrome | F84.2 |
| Encephalocele | Q01 |
| Microcephaly | Q02 |
| Down syndrome | Q90 |
| Edwards syndrome and Patau syndrome | Q91 |
| Other trisomies and partial trisomies of the autosomes,  not elsewhere classified | Q92 |
| Fragile X chromosome | Q99.2 |

**Table S2: Search String MEDLINE**

Search conducted in December/2024

Ovid MEDLINE® ALL <1946 to December 06, 2024>

| **1** | (((intellectual* or learning* or developmental*) adj3 (disab* or disord* or deficienc* or impair* or delay*)) or mental retardation* or Rett syndrome or Encephaloc?ele or Microcephal* or Down* syndrome or Trisomy 21 or Edwards* syndrome or Trisomy 18 or Patau* syndrome or Trisomy 13 or Fragile X chromosome).tw. |
| --- | --- |
| **2** | exp Intellectual Disability/ or Learning Disabilities/ or Developmental Disabilitites/ or Down Syndrome/ or Encephalocele/ or Microcephaly/ or Rett Syndrome/ or Trisomy 18 Syndrome/ or Trisomy 13 Syndrome/ or Fragile X Syndrome/ |
| **3** | (((cancer or breast or bowel or colon or colorectal or prostate or cervical or lung or gastric or stomach) and screening*) or PAP smear* or PAP test* or Papanicolaou or self-exam* or mammograph* or colonoscop* or coloscop* or Fecal Occult Blood Test* or Fecal Immunochemical Test* or early detection of cancer or (cancer adj5 detection)).tw. |
| **4** | Early Detection of Cancer/ or Mass Screening/ or Diagnostic Screening Programs/ or Mammography/ or Papanicolaou Test/ or Vaginal Smears/ or Colonoscopy/ |
| **5** | 1 or 2 |
| **6** | 3 or 4 |
| **7** | 5 and 6 |
| **8** | Limit 7 to yr="2013 - 2024" |

**Table S3: Search String Embase**

Search conducted in December/2024

Embase Classic+Embase <1947 to 2024 December 06>

| **1** | early cancer diagnosis/ or mass screening/ or mammography/ or Papanicolaou test/ or vaginal smear/ or cancer screening/ or colonoscopy/ |
| --- | --- |
| **2** | (((cancer or breast or bowel or colon or colorectal or prostate or cervical or lung or gastric or stomach) and screening*) or PAP smear* or PAP test* or Papanicolaou or self-exam* or mammograph* or colonoscop* or coloscop* or Fecal Occult Blood Test* or Fecal Immunochemical Test* or early detection of cancer or (cancer adj5 detection)).ti,ab. |
| **3** | exp intellectual impairment/ or developmental disorder/ or learning disorder/ or encephalocele/ or Rett syndrome/ or trisomy 13/ or trisomy 18/ or fragile X syndrome/ or Down syndrome/ or microcephaly/ or Edwards syndrome/ |
| **4** | (((intellectual* or learning* or developmental*) adj3 (disab* or disord* or deficienc* or impair* or delay*)) or mental retardation* or Rett syndrome or Encephaloc?ele or Microcephal* or Down* syndrome or Trisomy 21 or Edwards* syndrome or Trisomy 18 or Patau* syndrome or Trisomy 13 or Fragile X chromosome).ti,ab. |
| **5** | 1 or 2 |
| **6** | 3 or 4 |
| **7** | 5 and 6 |
| **8** | limit 7 to yr="2013 - 2024" |

**Table S4: Search String Web of Science**

Search conducted in December/2024

| **1** | TS=(((cancer or breast or bowel or colon or colorectal or prostate or cervical or lung or gastric or stomach) and screening*) or PAP smear* or PAP test* or Papanicolaou or self-exam* or mammograph* or colonoscop* or coloscop* or Fecal Occult Blood Test* or Fecal Immunochemical Test* or early detection of cancer or (cancer NEAR/5 detection)) |
| --- | --- |
| **2** | TS=(((intellectual* or learning* or developmental*) NEAR/3 (disab* or disord* or deficienc* or impair* or delay*)) or mental retardation* or Rett syndrome or Encephaloc?ele or Microcephal* or Down* syndrome or Trisomy 21 or Edwards* syndrome or Trisomy 18 or Patau* syndrome or Trisomy 13 or Fragile X chromosome) |
| **3** | #1 AND #2 |
| **4** | #1 AND #2 and 2025 or 2024 or 2023 or 2022 or 2021 or 2020 or 2019 or 2018 or 2017 or 2016 or 2015 or 2014 or 2013 (Publication Years) |
